# Designing and Development of FRET-Based Nanosensor for Real Time Analysis of N-Acetyl-5-Neuraminic Acid in Living Cells

**DOI:** 10.3389/fnut.2021.621273

**Published:** 2021-05-31

**Authors:** Ruphi Naz, Mohammad K. Okla, Urooj Fatima, Mohd. Mohsin, Walid H. Soufan, Ibrahim A. Alaraidh, Mostafa A. Abdel-Maksoud, Altaf Ahmad

**Affiliations:** ^1^Department of Botany, Faculty of Life Sciences, Aligarh Muslim University, Aligarh, India; ^2^Department of Botany and Microbiology, College of Science, King Saud University, Riyadh, Saudi Arabia; ^3^Department of Biosciences, Jamia Millia Islamia, New Delhi, India; ^4^Department of Plant Production, Faculty of Food and Agriculture Sciences, King Saud University, Riyadh, Saudi Arabia; ^5^Department of Zoology, College of Science, King Saud University, Riyadh, Saudi Arabia

**Keywords:** fluorescence resonance energy transfer, sialic acid, nanosensor, fluxomics, NeuAc

## Abstract

*N*-acetyl-5-neuraminic acid (NeuAc) plays crucial role in improving the growth, brain development, brain health maintenance, and immunity enhancement of infants. Commercially, it is used in the production of antiviral drugs, infant milk formulas, cosmetics, dietary supplements, and pharmaceutical products. Because of the rapidly increasing demand, metabolic engineering approach has attracted increasing attention for NeuAc biosynthesis. However, knowledge of metabolite flux in biosynthetic pathways is one of the major challenges in the practice of metabolic engineering. So, an understanding of the flux of NeuAc is needed to determine its cellular level at real time. The analysis of the flux can only be performed using a tool that has the capacity to measure metabolite level in cells without affecting other metabolic processes. A Fluorescence Resonance Energy Transfer (FRET)-based genetically-encoded nanosensor has been generated in this study to monitor the level of NeuAc in prokaryotic and eukaryotic cells. Sialic acid periplasmic binding protein (SiaP) from *Haemophilus influenzae* was exploited as a sensory element for the generation of nanosensor. The enhanced cyan fluorescent protein (ECFP) and Venus were used as Fluroscence Resonance Energy Transfer (FRET) pair. The nanosensor, which was termed fluorescent indicator protein for sialic acid (FLIP-SA), was successfully transformed into, and expressed in *Escherichia coli* BL21 (DE3) cells. The expressed protein of the nanosensor was isolated and purified. The purified nanosensor protein was characterized to assess the affinity, specificity, and stability in the pH range. The developed nanosensor exhibited FRET change after addition to NeuAc. The developed nanosensor was highly specific, exhibited pH stability, and detected NeuAc levels in the nanomolar to milimolar range. FLIP-SA was successfully introduced in bacterial and yeast cells and reported the real-time intracellular levels of NeuAc non-invasively. The FLIP-SA is an excellent tool for the metabolic flux analysis of the NeuAc biosynthetic pathway and, thus, may help unravel the regulatory mechanism of the metabolic pathway of NeuAc. Furthermore, FLIP-SA can be used for the high-throughput screening of *E. coli* mutant libraries for varied NeuAc production levels.

## Introduction

The *N*-acetyl-5-neuraminic acid (NeuAc) is the most common member of the sialic acid (SA) family (1, 2). It is engaged in various metabolic functions in bacteria and animals. In humans, NeuAc regulates biological recognition, immunological processes, several hormonal responses, cell–cell communication, transmission of neuronal signals, etc. (3). Disturbances in the metabolism of NeuAc in humans results in severe pathological conditions (4). As NeuAc has a negative charge, it contributes to the biophysical properties of living systems. This negative charge causes charge repulsion and prevents the undesirable contacts of cells in the blood circulation (5, 6). It has also been reported that SA affects fertilization (7). Moreover, NeuAc is involved in glycoconjugate recognition based on biospecific activities (8). It has been reported that the sialyl oligosaccharides on the surface of cells act as a receptor element for the influenza virus, and bacterial toxins (9). Thus, novel therapeutic (10) and diagnostic materials for the treatment of cancer, nerve-related diseases, autoimmune diseases, and other infectious diseases (11) are being developed based on the multifunctionality of NeuAc. Potent antiviral drugs such as zanamivir and oseltamivir have also been synthesized from NeuAc (12, 13). Most importantly, it is an important component of the human milk oligosaccharide (HMO) family that helps in the improved development of infant (14). The SA-containing HMO (sialyllactoses) is involved in the promotion of probiotic proliferation and in muscle and liver development in newborn babies, as well as in the optimization of brain metabolism (15).

According to the Sialic Acid Market Research Report (16), the annual growth rate of NeuAc demand in the global market is expected to increase by 12.19% by 2024. Recently, it has been certified as a food additive by various food safety authorities in the Europe, US and China (17, 18). In addition NeuAc has huge applications in the pharmaceutical, cosmetic, food additive, and medical fields. Traditionally, NeuAc is obtained via extraction from natural resources such as cubilose, eggs, milk, and casein and by chemical synthesis from α-(bromomethyl) acrylic acid. However, the concentration of extracted NeuAc from natural sources is very low. Further, several expensive and toxic metals are required as catalysts for the synthesis of NeuAc chemically, which increases its production cost.

In contrast with the procurement of NeuAc using chemical as well as extraction methods, the NeuAc production via a biological method will certainly be much cheaper (19). In recent years, the metabolic engineering approach has been used for the production of a large number of substances, including NeuAc (2, 19, 20). However, the production of NeuAc is still not cost-effective because regulatory elements of its biosynthetic pathway are not identified that is a major bottlenecks for increased production of NeuAc. The identification of regulatory step can be carried out via a fluxomics analysis of the metabolic pathway. However, a tool that measures the level of the metabolite non-invasively in living systems is needed for this fluxomics analysis. Genetically encoded Fluorescence Resonance Energy Transfer (FRET)-based nanosensors offer such a tool for a large number of metabolites. These nanosensors are considered ideal for the understanding the elements of regulation in a metabolic pathway because they allow the monitoring of the metabolite even at the single cell level in a non-invasive manner and with better spatial resolution (21, 22). Nanosensors for glutamate (23), arginine (22), catechin (24), ajamalicin (25) and for many other metabolites have been developed. The working principle of these nanosensors is based on the FRET phenomenon, which is a physical process in which distance-dependent non-radiative transfer of energy takes places from the donor to the acceptor fluorophore through intermolecular dipole–dipole coupling. A metabolite-binding protein acts as a sensory domain. This protein is “sandwiched” between the donor and acceptor fluorophores, and binding of the metabolite to the sensory domain results in a change in the emission intensities of both the fluorophores. Ratio of emission intensities of these fluorophores is taken to measure the level of the metabolite. A genetically encoded FRET-based nanosensor taking fluorescent indicator protein for sialic acid (FLIP-SA), was designed and developed in this study for the measurement of NeuAc in living systems non-invasively. This sensor is cost-effective and can be useful for the metabolic flux analysis of NeuAc in its metabolic network, to identify the regulatory step of the NeuAc biosynthetic pathway. Furthermore, FLIP-SA can be used for the high-throughput screening of *E. coli* mutant libraries for varied NeuAc production levels.

## Materials and Methods

### Designing and Construction of NeuAc Nanosensor

A Protein Databank search for a suitable SA- binding protein resulted in the identification of a periplasmic binding protein (SiaP) of Haemophilus influenzae (strain KW20/ Rd) that binds to NeuAc specifically. The crystal structure of SiaP in both the open and closed form has been published previously (26, 27). The conformational change of the SiaP protein was confirmed (PDB-3B50; resolution, 1.4 Å). This protein was used as the NeuAc sensing part for nanosensor construction. The HI0146 gene was found to encode the SiaP protein. The sequence of this gene was retrieved from the National Center for Biotechnology Information (NCBI). Initial nucelotide sequences encoding the signal peptides were identified. Amplification of the HI0146 gene without the signal peptide sequence was carried out through PCR from the genomic DNA of H. influenzae. KpnI restriction endonuclease sites were added using the following primers: forward, 5′-CGGGGTACCATTATGACTTGAAATTCGGTA–′3' and reverse, 5′-CGGGGTACCGTGTTGGTGGTGGTGGTGGGA−3′. During primer design, the stop codon was eliminated to overcome the problem of truncation. The fragment amplified from the SiaP gene was ligated between the enhanced cyan fluorescent protein (ECFP)/Venus, creating the construct of ECFP-SiaP-Venus, resulting in pGEMT-ECFP-SiaP-Venus. Validation of the cloned product was carried out through restriction digestion and sequencing. The ECFP-SiaP-Venus was then shuttled from the pGEMT-easy vector to the pRSET-B expression vector (Invitrogen, USA), resulting in the pRSET-ECFP-SiaP-Venus nanosensor. The pRSET-ECFP-SiaP-Venus nanosensor was termed FLIP-SA nanosensor that has been transformed into the BL21 (DE3) strain of *Escherichia coli* through electroporation. Furthermore, ECFP-SiaP-Venus was shuttled to the yeast expression vector (pYES-DEST52) using the Gateway technique (Invitrogen, USA). First, an entry clone, pDONR-ECFP-SiaP-Venus, was generated using a BP recombination reaction between the ECFP-SiaP-Venus construct and the pDONR22 vector. Subsequently, pYES-DEST52-ECFP-SiaP-Venus was generated as expression clone through an LR-mediated reaction.

### Expression, Isolation, and Purification of FLIP-SA Protein

*Escherichia coli* BL21 (DE3) cells carrying the nanosensor construct were grown on Luria–Bertani (LB) medium containing ampicillin at 20°C for 24 h. Induction of the nanosensor protein was carried out by adding 0.5 mM Isopropyl β-D-1-thiogalactopyranoside, Isopropyl β-Dthiogalactoside (IPTG) (Fermentas, Germany). After induction, cells were kept for 48 h in the dark at 16°C with continuous shaking. The cells were then harvested and centrifuged at 6,500 × g at 4°C for 30 min, for the isolation of the nanosensor protein. The cell pellet was resuspended in chilled Tris-HCl buffer (20 mM, pH 7.2). An ultrasonicator (Sonics, USA) was used for bacterial cells lysis, followed by centrifugation and filtration of the lysate, to remove cell debris. The isolated protein fraction was collected and applied to nickel-aminotriacetic acid (Ni-NTA) column (BioRad, CA, USA) for His-tag-mediated purification of the nanosensor protein. After washing of the column, elution of the sensor protein was carried out by applying elution buffer (20 mMTris-Cl, pH 8.0, 250 mM imidazole). The eluted protein was incubated at 4°C for 12 h, for proper folding.

### Characterization of FLIP-SA Nanosensor

#### pH Stability Testing

Three buffer systems [phosphate-buffered saline (PBS), triethanolamine-buffered saline (TBS), and 3-(N Morpholino) Propanesulfonic Acid (MOPS); all 20 mM] with a pH range of 5.5–8.0 were used to test the pH stability of the nanosensor. Among the buffers tested, PBS was deemed suitable for further analysis because the least variation in FRET ratio (535/485 nm) was observed when the sensor protein was treated with PBS buffer at different pH values. The ratio of the emission intensity of Venus/ECFP with reference to different pH values and buffer systems was recorded using a microplate reader (Synergy H1, Biotek, USA).

#### Spectral Analysis

The emission spectra of the FLIP-SA nanosensor were recorded using a spectrofluorometer (LS50B; Perkin Elmer, USA). The nanosensor protein was diluted using PBS buffer (20 mM, pH 7.0). For monitoring the fluorescence emission spectra, the ECFP was excited and then recording of the emission intensities (450–600 nm) was carried out before and after the addition of NeuAc.

#### Specificity and Affinity Analysis

The specificity of FLIP-SA was analyzed using various related sugars (glucose, sucrose, galactose, and lactose). 1.0 μM and 1.0 mM of these sugars were added to the diluted nanosensor protein, and the ratio of the emission intensities of Venus and ECFP was measured. A Synergy H1-make microplate reader equipped with a 430/20 nm filter set (for the excitation of ECFP) and filter sets of 485/20 nm (for ECFP emission) and 535/25 nm (for Venus emission) were used. Titration of the nanosensor protein was carried out using concentrations of NeuAc of 1.0 nM to 1.0 M, to calculate the affinity of FLIF-SA and binding constant (*K*_*d*_) was calculated according to Ameen et al. (22).

### Real-Time Monitoring of N-Acetyl-5-Neuraminic Acid in Bacterial Cells Using FLIP-SA

*E. coli* BL21 (DE3) cells containing FLIP-SA were incubated in LB medium at 20°C overnight, followed by the induction of the expression of the sensor protein using 0.5 mM IPTG. Subsequently, the bacterial cells were grown at 16°C for 48 h in a shaking incubator and then stored overnight at 4°C. The bacterial cells were pelleted down using a high-speed cooling centrifuge (Eppendorf, USA), resuspended in M9 medium. The pH of the medium was maintained at 7.0. The cells were starved for 3 h. A standard amount of bacterial cell culture (180 μl) was poured into a 96-well microplate in triplicate. NeuAc (1.0 mM; 20 μl) was then added to each well. The emission intensities of the donor and acceptor fluorophores were recorded at 485/20 nm and 535/25 nm, respectively, after excitation of the donor fluorophore at 430/20 nm using the microplate reader and filter sets mentioned above. The injection module of the microplate reader was used to monitor the accumulation rates. To check the specificity of FLIP-SA, *in vivo* experiments were also carried out by adding 0.1 and 1.0 mM glucose, sucrose, galactose, and lactose separately in place of NeuAc. All experiments were conducted in triplicate and independently. Imaging of the expressed sensor in the bacterial cells was carried out using a confocal microscope (Leica, Germany).

### Real-Time Monitoring of N-Acetyl-5-Neuraminic Acid in Yeast Cells Using FLIP-SA

N-Acetyl-5-neuraminic acid monitoring was also carried out in an eukaryotic system. For this purpose, the yeast strain BY4742 was used for the expression of the FLIP-SA nanosensor. Synthetic Defined (SD) medium was used for the culture of yeast cells for 3–6 days. pYES-DEST_ECFP-SiaP-Venus was introduced into the cultured yeast. Subsequently, 2% dextrose (carbon source) and 1% galactose (inducer) were added to the SD medium. The monitoring of NeuAc and imaging of FLIP-SA in yeast cells was carried out using a Confocal Laser Scanning Microscope (CLSM;) (DMRE; Leica, Wetzlar, Germany) equipped with a confocal head (TCS SPE; Leica, Wetzlar, Germany) and a 63× oil-immersion objective. The dual emission intensity ratio was recorded using the LAS-AF software (Leica, Wetzlar, Germany) with 435/20 excitation and two emission filters (485/40 for CFP and 535/30 for YFP) and a neutral density filter (1 or 5% transmission) on the excitation port. Ethanol-washed poly l-lysine-coated cover slips were used to fix the yeast cells using superglue. NeuAc was slowly added to the bath solution using a nanoliter pipette positioned closely to the yeast cells in focus. The FRET sensitized emission tool without background subtraction of the LAS-AF software was used to record and display the data. The data were processed and displayed using the LAS-AF software and Adobe Photoshop. Images were acquired from single cells and multiple cells by selecting the region of interest.

### Creation of Affinity Mutants of FLIP-SA

To increase the detection limit of the FLIP-SA nanosensor, several affinity mutants were also created using a site-directed mutagenesis kit (Stratagene, USA). The structure of SiaP was taken from the a protein database (PDB-3B50) for the identification of the amino acid residues of the NeuAc-binding pocket. Point mutations were created by the modification of the amino acids of the NeuAc-binding pocket, to assess the changes in the affinity of the nanosensor. Sensor variants were developed by replacing aspartate 49 with lysine (D49K), arginine 127 with aspartic acid (R127D), and glutamate 67 with asparagine (E67N). The developed mutants were expressed, purified, and characterized as explained above.

### Statistical Analysis

Statistical analyses and graphs were prepared using the SigmaPlot version 11 software (Systat Software). Mean values and standard errors were calculated from three independent replicates. An analysis of significance was performed using Students' *t*-test. Significance was set at *P* < 0.05.

## Results

### Designing and Develoment of FLIP-SA Nanosensor

**Figure 1:**
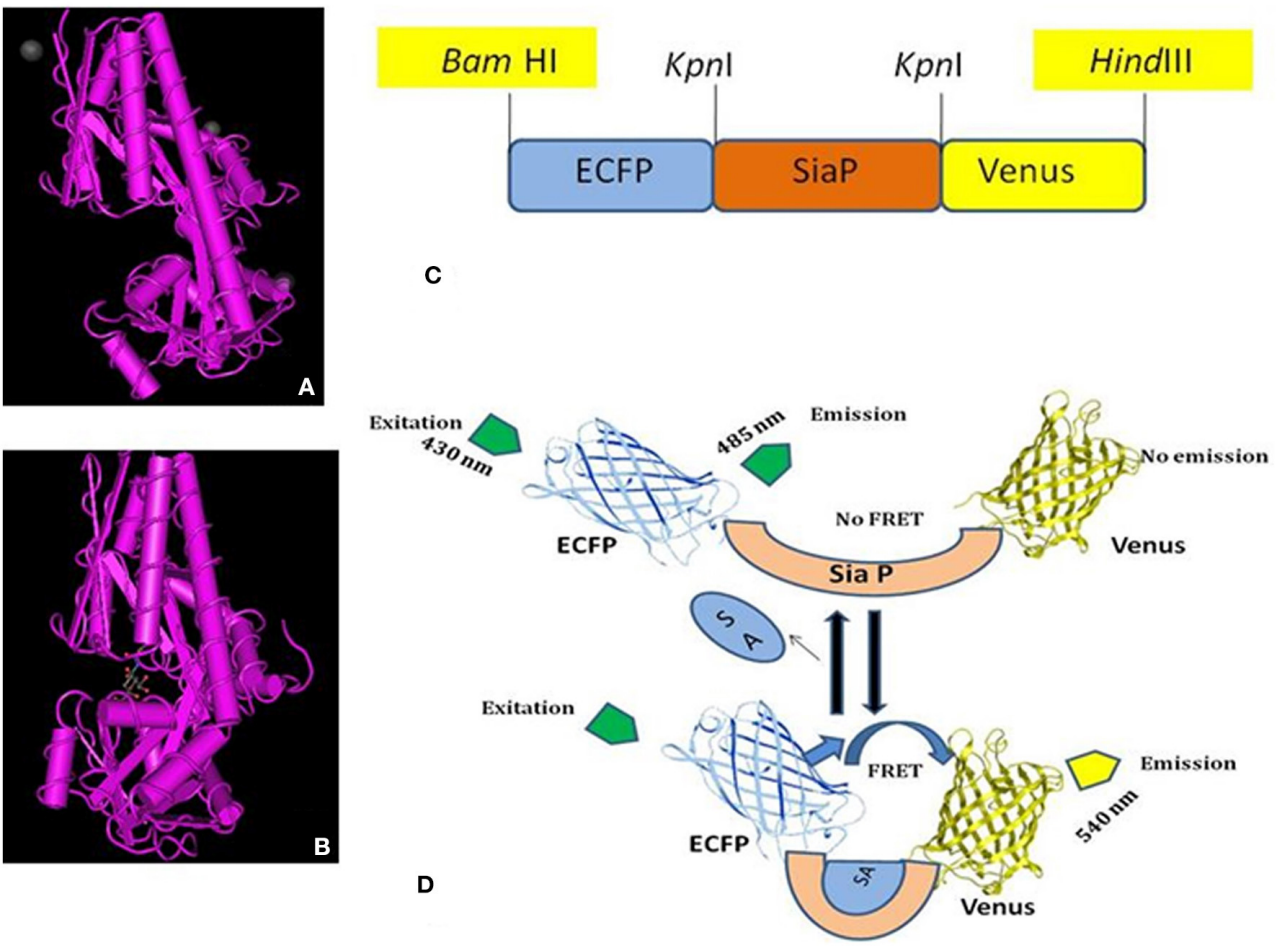
NeuAc nanosensor. **(A)** Open form of SiaP protein, **(B)** Closed form of SiaP protein (26, 27), **(C)** Diagram represents the restriction sites of ECFP-SiaP-Venus construct, **(D)** General demonstration of working of FLIP-SA. Addition of NeuAc with the sensory domain (SiaP) brings the ECFP and Venus closure, resulting the FRET.

The FLIP-SA nanosensor was developed to monitor NeuAc dynamics in real time in prokaryote and eukaryote systems non-invasively. The NeuAc-binding protein, SiaP, obtained from *H. influenzae* was employed as a ligand-binding domain. The SiaP protein holds two domains that form a well-characterized pocket. At the point where NeuAc ties to SiaP, the pocket comes closer and the two domains twist at a hinge region (28, 29) (Figure 1A). The two structure types (open, A; and close, B) of SiaP are shown in Figures 1A,B (28). The GFP variants, ECFP and Venus, together with the SiaP protein, were used for the construction of the nanosensor, to determine the changes in NeuAc levels in real time. The N and C termini of the SiaP protein were ligated to ECFP and Venus, respectively (Figure 1C). Figure 1D depicted the working mechanism of the sensor. A conformational change was occurred after the binding of the sialic acid to the sensory part of SiaP. As a result, the two fluorophores came closer to each other, and energy is transferred non-radiatively from ECFP to Venus, thus yielding a FRET change. In the absence of ligand, there was no significant change in the FRET. FRET is highly efficient if the donor and acceptor fluorophores are in close proximity (<10 nm).

Amplified gene of the ECFP, HI0146 and Venus were successfully ligated together at *Kpn*I restriction endonuclease site, generating ECFP-SiaP-Venus that was cloned in pGEM®-Teasy. The construct was transformed to *E. coli* DH10B. Restriction digestion of the pGEM®-Teasy_ECFP-SiaP-Venus construct using *Bam*HI and *Hin*dIII restriction endonuclease enzymes resulted in the expected fragments of 3.15 and 2.3 kb. It validated the successful development of the ECFP-SiaP-Venus construct (Supplementary Figure 1). Analysis of the pRSET-B-ECFP-SiaP-Venus on agarose gel revealed that the size of the construct is 5.3 kb that is the expected size of the construct (Supplementary Figure 2). Restriction digestion of the pRSET-B-ECFP-SiaP-Venus using *Kpn*I restriction endonuclease showed two bands of 4.3 kb (pRSET-B- ECFP-Venus) and 0.920 kb (SiaP) (Supplementary Figure 2). It validated the successful generation of the nanosensor. Further gene sequencing of the ECFP-Venus-HI0146 construct showed its fidelity (Supplementary Figures 3–5). The construct was named as FLIP-SA (Fluorescent Indicator Protein for Sialic Acid). Supplementary Figure 6 showed the schematic representation of FLIP-SA map in cloning and expression vectors. The nanosensor protein was expressed in bacterial cells. The expressed protein was isolated and purified using the property of the His6-affinity tag of pRSET-B vector to bind with the Ni-NTA beads.

### Characterization of the Nanosensor

**Figure 2:**
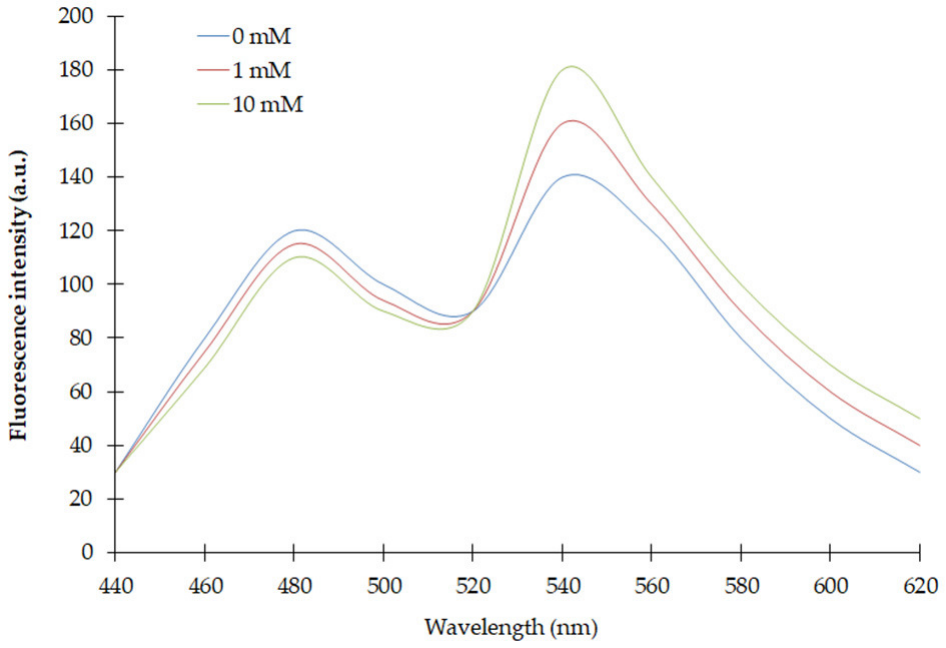
Spectral analysis of FLIP-SA. Change in fluorescence emission was observed from 440 to 620 nm using fluorescence spectrophotometer without NeuAc (control) and with NeuAc (1 and 10 mM). 0.20 mg/ml sensor protein was taken.

**Figure 3:**
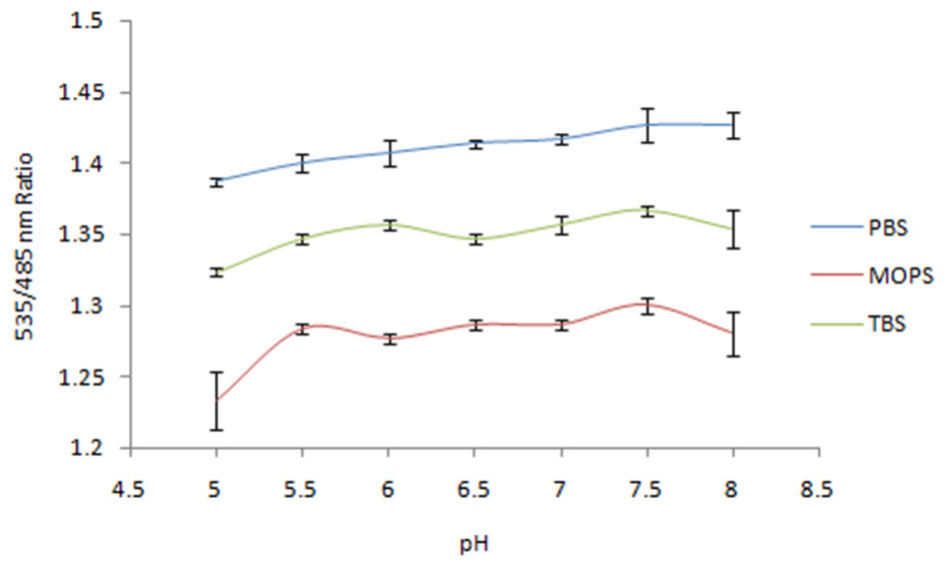
Sensitivity analysis of nanosensor toward pH in different buffer systems. The data are the average of three replications. Vertical bars show the standard errors. 0.20 mg/ml sensor protein was taken.

Spectral analysis of the purified FLIP-SA showed that addition of NeuAc resulted in the shift in the emission intensity at 480 and 540 nm compared with the control (without addition of NeuAc). The emission intensity at 540 nm increased, and that at 480 nm decreased (Figure 2). The analysis of the FRET ratio of the FILP-SA nanosensor at different pH ranges and using three buffer systems showed no significant change in the FRET ratio of the nanosensor from pH 5.5 to 8.0. Among the different buffers tested, PBS was found to be the most suitable, as the FRET ratio was the highest in this buffer system compared with the MOPS and TBS buffers (Figure 3). Thus, the PBS buffer at pH 6.5 was selected for further experiments.

**Figure 4:**
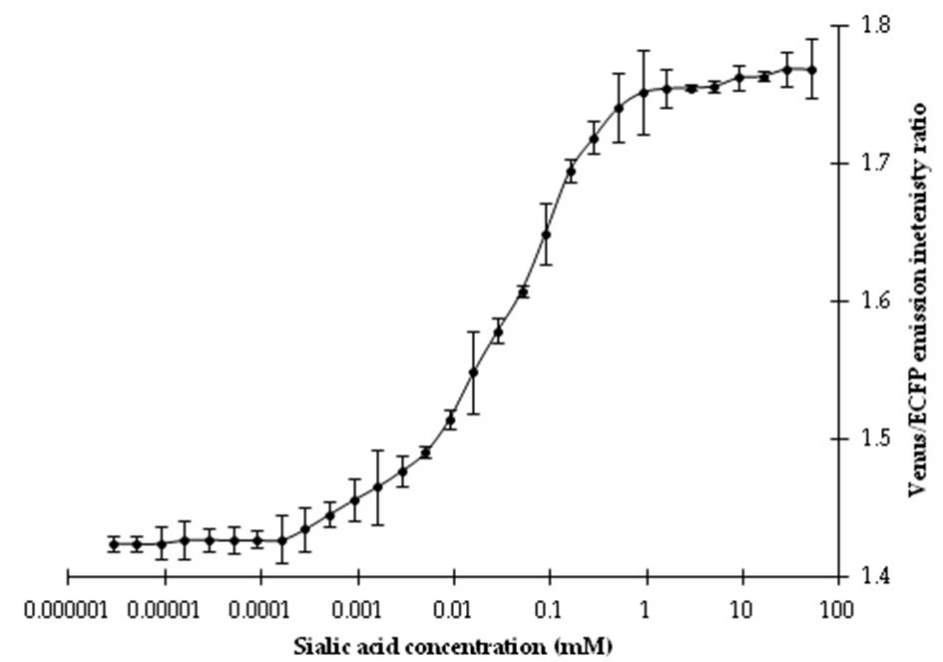
Titration analysis of the nanosensor at different concentrations of NeuAc. The data are the average of three replications. Vertical bars show the standard errors. 0.20 mg/ml sensor protein was taken.

Affinity titration of this nanosensor showed that a saturation curve of FRET ratio was formed by the addition of different concentrations of NeuAc (1 nM−1 M). The addition of NeuAc resulted in a change of the emission intensities of the Venus and ECFP fluorophores of the purified nanosensor protein. Significant changes in the FRET ratio were reported from 1 μM to 1 mM NeuAc, as shown by the sigmoid curves (Figure 4). The calculated affinity (*K*_*d*_) of the FLIP-SA nanosensor to NeuAc was ~157 μM. In the current experiment, the *K*_*d*_ was calculated based on changes in the FRET ratio together with the conformational changes in the chimeric sensor protein.

**Figure 5:**
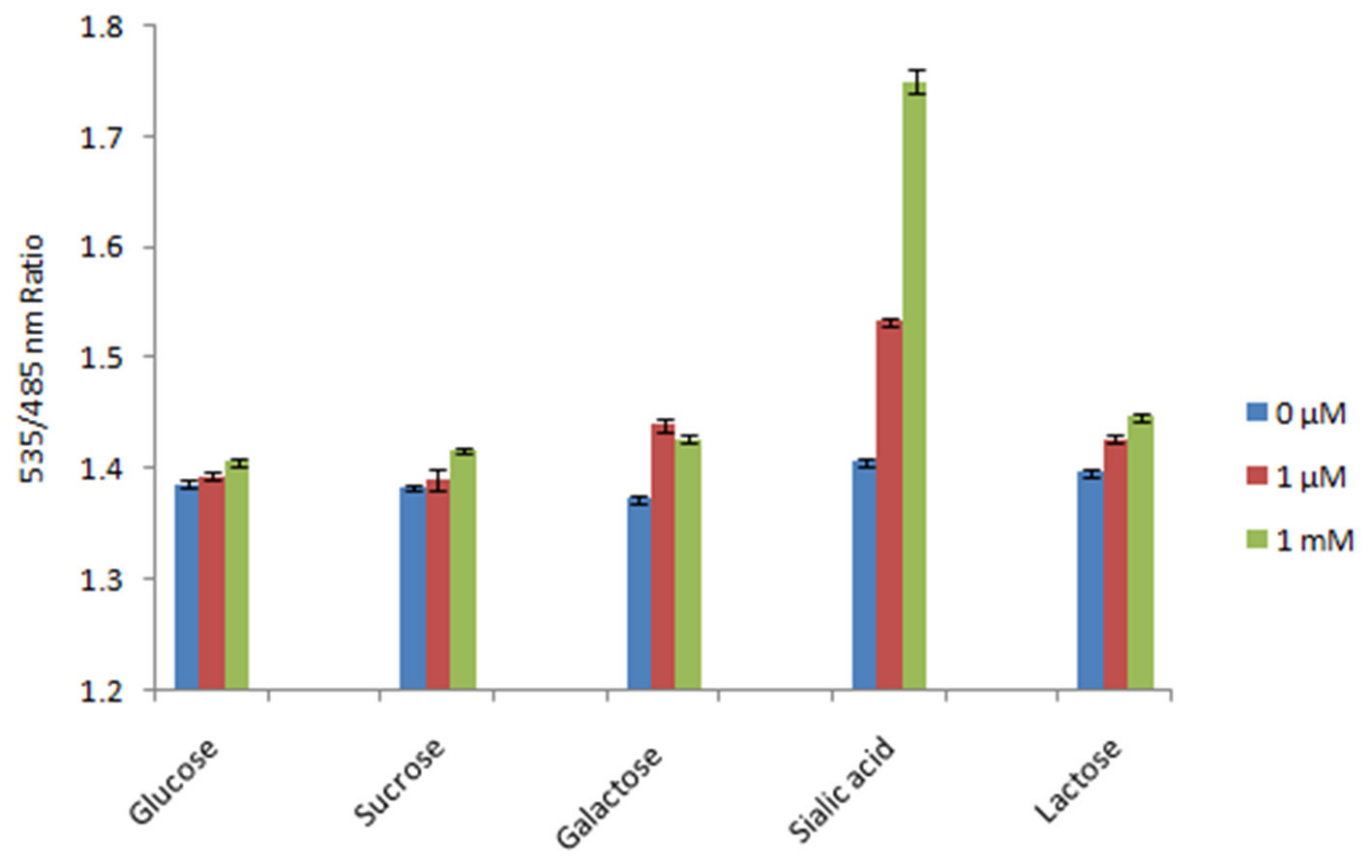
Specificity analysis of FLIP-SA. The specificity of the sensor was recorded based on the changes in FRET ratio after the addition of NeuAc and other sugars at 0 μM, 1.0 μM, and 1.0 mM. The data are the average of three replications. Vertical bars show the standard errors. 0.20 mg/ml sensor protein was taken.

The specificity test of the FLIP-SA nanosensor showed that the addition of other monosaccharides (glucose, sucrose, galactose, and lactose) did not change the FRET ratio of the nanosensor protein significantly. However, the addition of 1.0 mM NeuAc triggered a significant increase in the FRET ratio (~1.753). Thus, the FLIP-SA sensor showed a maximum FRET change toward NeuAc, which suggests that the FLIP-SA sensor is very specific for NeuAc (Figure 5).

**Figure 6:**
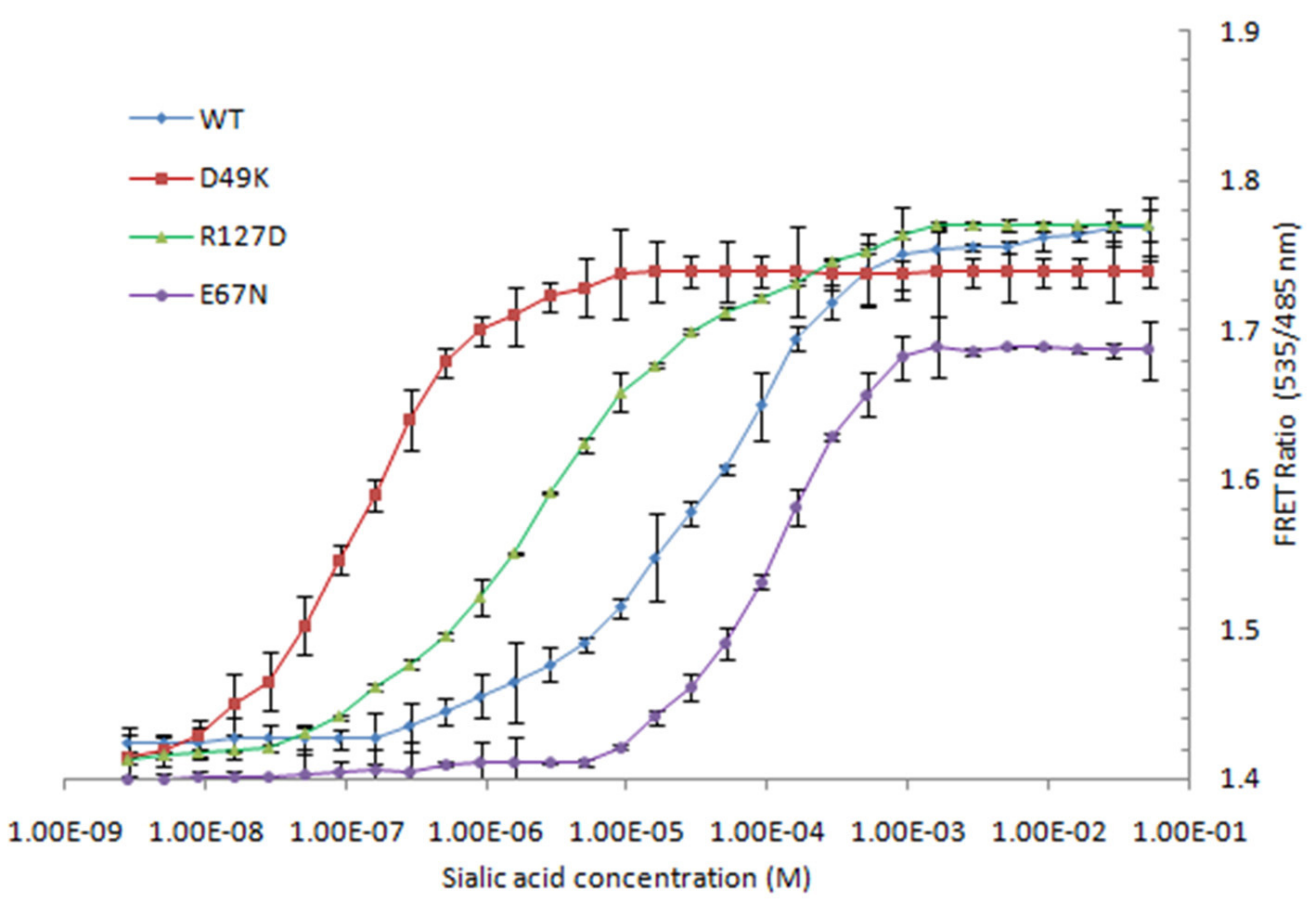
Titration analysis of various affinity mutants (D49K, R127D, and E67N) FLIP-SA. The study was conducted with three replicates independently. Vertical bars show the standard errors. 0.20 mg/ml sensor protein was taken.

To increase the NeuAc detection range of the FLIP-SA, three affinity mutants of FLIP-SA were created through site-directed point mutagenesis. These nanosensor variants showed different binding affinities across a wide concentration range, from 1.0 nM to 1.0 mM. The calculated *K*_*d*_ values of the wild-type protein and the D49K, R127D, and E67N affinity mutants were 157 μM, 13 nM, 510 nM, and 600 μM, respectively. Maximum changes in FRET ratio were observed for the D49K and R127D mutants (Table 1, Figure 6).

**Table 1:** NeuAc-binding affinity of the different mutants.

**Nanosensors^a^**	**Generated mutants**	***K_***d***_***	**Detection range^b^**	**ΔRmax^§^**
Flip-SA 157	Wild-type protein	157 μM	1.58 μM−1.56 mM	0.25
Flip-SA 13	D49K	13 nM	1.0 nM−49.5 μM	0.32
Flip-SA 510	R127D	510 nM	80 nM−96.7 μM	0.34
Flip-SA 600	E67N	600μM	24 μM−10 mM	0.14

a *The nanosensor mutant name was based on the K_d_value*.

b *The quantification range fell between 10 and 90% saturation of the sensors*.

§ *Maximal change in the FRET ratio of the sensor*.

### Real-Time Measurement of NeuAc in Living Cells Using FLIP-SA

**Figure 7:**
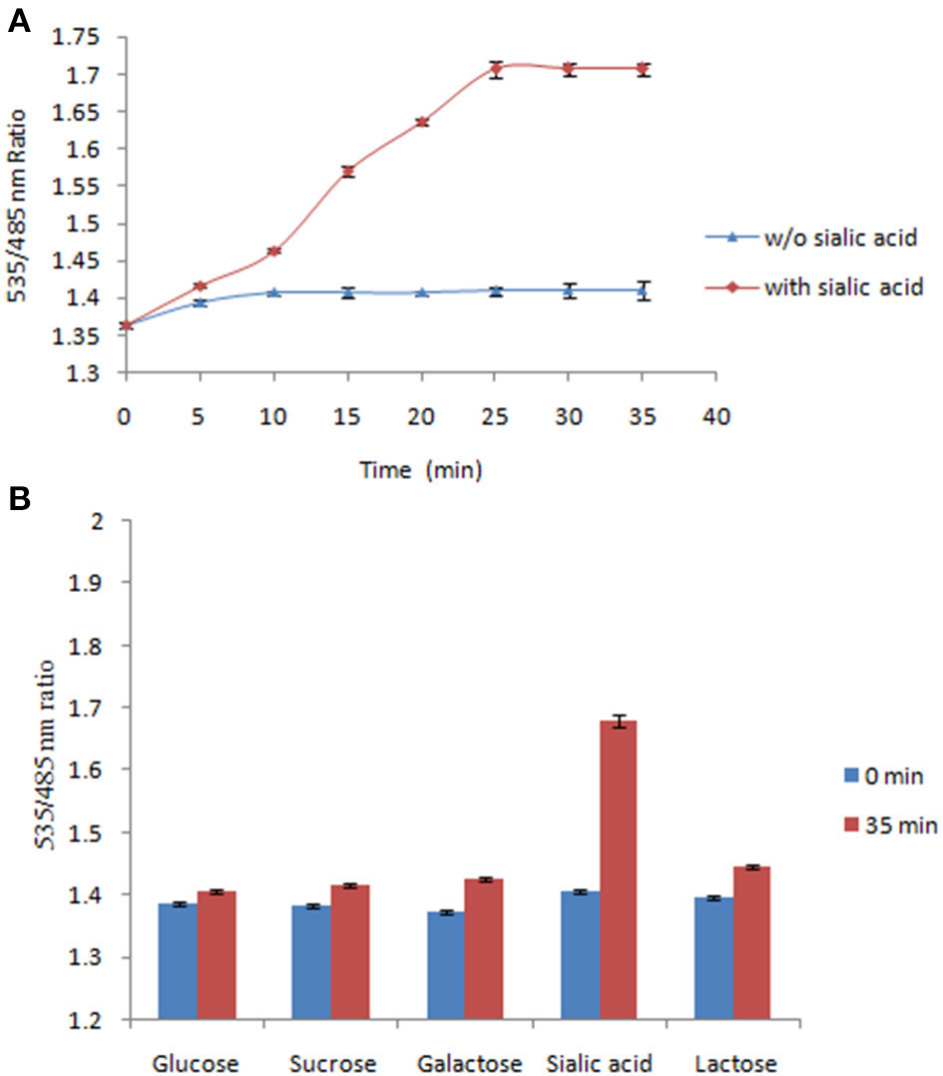
Measurement of NeuAc in bacteria using FLIP-SA. **(A)** Measurement of NeuAc for 35 min after adding 1.0 mM NeuAc to bacterial cells expressing FLIP-SA (red circles). Measurement of NeuAc for 35 min without adding NeuAc (blue circles). **(B)** Measurement of the FRET ratio of bacterial cells expressing FLIP-SA after incubation with different sugars at a concentration of 1.0 mM for 35 min. The data are the average of three replications. Vertical bars show the standard errors.

The FLIP-SA sensor was used to measure NeuAc in live bacterial cells. It was shown that the addition of NeuAc at a concentration of 1.0 mM to FLIP-SA-expressing bacterial cells resulted in a significant incerase in the FRET ratio compared with the FRET ratio obtained in the absence of NeuAc. This increase in the FRET ratio was observed 5 min after the addition of NeuAc; subsequently, the saturation was recorded at 35 min. The Venus/ECFP emission intensities were recorded up to the saturation phase (Figure 7A). The present results demonstrated that the addition of exogenous NeuAc to FLIP-SA-expressing bacterial cells leads to a response from the FLIP-SA sensor. Saturation within 30–35 min at 1.0 mM NeuAc led to the storage of NeuAc inside bacterial cells. Authenticity was confirmed by the recording of a major FRET ratio change in the presence of NeuAc compared with other related compounds (Figure 7B). These results confirmed the specificity of FLIP-SA toward NeuAc in bacterial cells in an *in vivo* environment.

**Figure 8:**
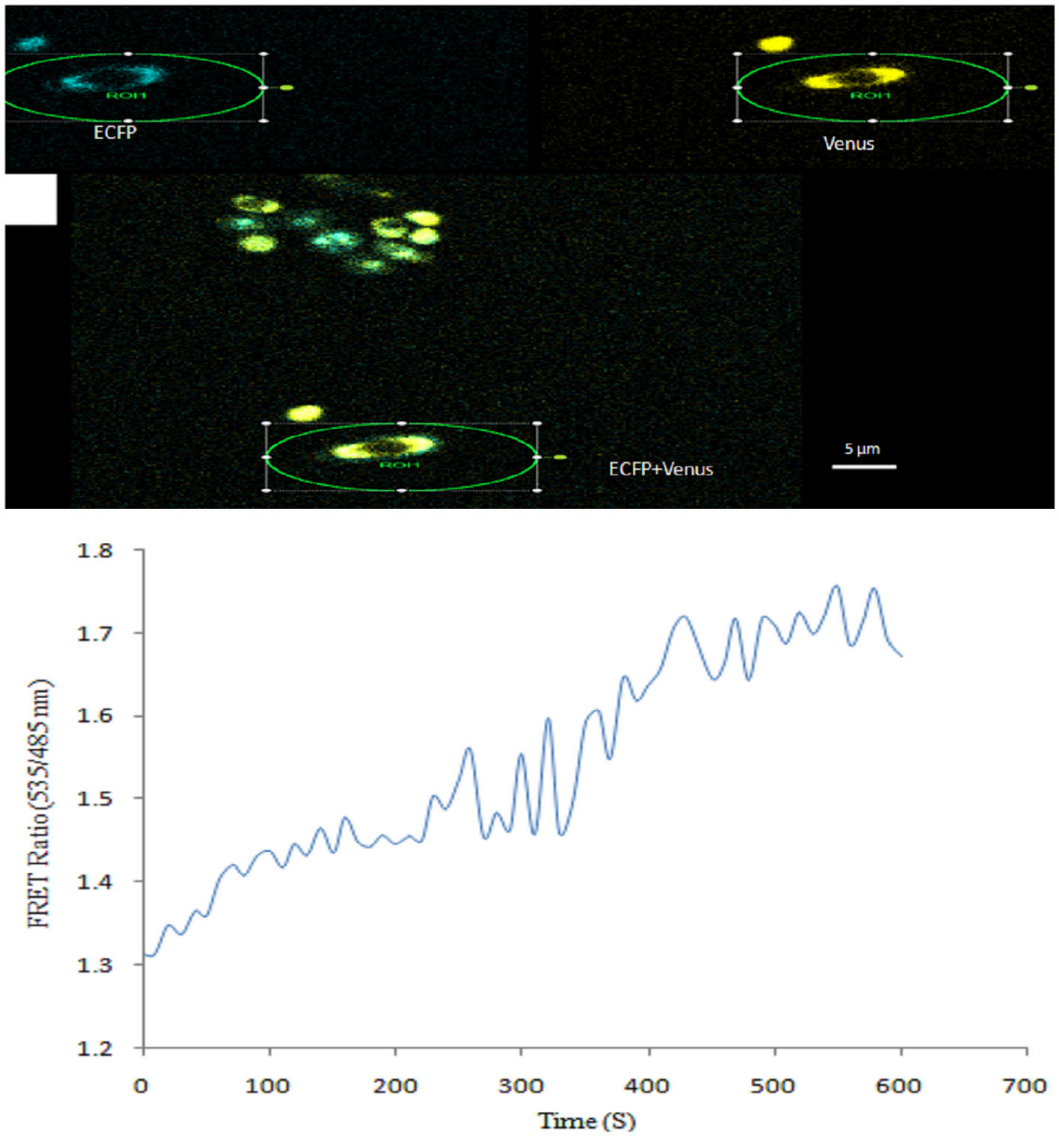
Measurement of NeuAc in yeast cells using FLIP-SA. **(A)** Yeast cells showing expression of the FLIP-SA sensor in the cytosol (scale bar, 10 μm). **(B)** Time-course (0–610 s) monitoring of the level of NeuAc in yeast cells by FLIP-SA after the addition of NeuAc.

Real-time monitoring of NeuAc was also carried out in yeast cells expressing FLIP-SA. Imaging of FLIP-SA-transformed yeast cells confirmed the presence of the sensor protein in the cytosol. The addition of NeuAc (1.0 mM) to the FLIP-SA-expressing yeast cells triggered changes in the emission intensity of ECFP and Venus. The emission intensity ratio of Venus/ECFP was monitored for 600 s and found to have increased from 1.299 at 0 s to 1.760 at 510 s after the addition of NeuAc (Figure 8).

## Discussion

NeuAc is the most abundant of the SA and has wide commercial importance. The synthesis of many novel medicinal drugs and analytical systems for influenza viruses is based on NeuAc (26). Neuro developmental research studies have reported that NeuAc is an important factor in the role of nutrients in brain growth and development in infants (27, 30). As a result, NeuAc is being documented as an important component of milk-based products for infants. NeuAc is also used in the manufacture of cosmetics (16). Given the high requirement for NeuAc in the medical, functional infant foods, and cosmetics fields, the worldwide market demand for NeuAc is increasing continuously. The metabolic engineering method has revolutionized the biological synthesis of metabolites in recent years. Efforts have also been made toward the biosynthesis of NeuAc using biological processes (2, 31, 32). However, the production of NeuAc using the metabolic engineering approach is still not cost-effective because of the complexity of pathway (20) and its regulatory mechanism is not fully understood, as the tools for the metabolic flux analysis of this pathway are lacking.

The present study offers a genetically encoded FRET-based nanosensor (FLIP-SA) that can be used to perform metabolic flux analyses through the real-time measurement of NeuAc in living cells non-invasively. Such type of nanosensors have become an alternative and powerful analytical tool to visualize and analyze the presence of small molecules, ion fluxes, signaling components, and metabolites, or for sensing the environmental conditions in real time with high spatial and temporal resolution (33, 34). A nanosensor is a fusion protein that consists of a central binding domain linked by two fluorescent proteins. The optical readout of the sensor is based on FRET that changes upon binding with ligand. Due to the binding of ligand, a conformational change in the protein is a proxy for the ratiometric measurement of bioactive molecules. Although the concept of FRET has been theoretically explored for a decade but in recent year it has become applied successfully in sensing research (34, 35). FRET is a distance dependant radiationless transfer of energy from the donor to acceptor molecules.

One of the requirements for the development of the FRET sensor is the availability of a protein that changes its conformation upon binding to the ligand of the interest. The sialic acid periplasmic binding protein of *Haemophilus influenzae* (strain KW20/Rd) in the presence of NeuAc forms deep interaction by its carboxylate group (28), and shows conformation change like “venus-fly-trap” manner. In the present study, we successfully constructed the NeuAc nanosensor (FLIP-SA) by using periplasmic binding domain of sialic acid protein (SiaP) along with variants of green fluorescent proteins (ECFP and Venus) to monitor the NeuAc fluxes in prokaryote as well as in eukaryote. The construct (ECFP-SiaP-Venus sequences) was sub-cloned in expression vector. Similar to our results a number of nanosensors were developed by using fluorescent protein variants (36, 37). Expression of the FLIP-SA was monitored after the transformation of construct (pRSET-ECFP-SiaP-Venus) into the *E. coli* BL21 (DE3). Spectral analysis of purified sensor protein demonstrated an enhancement in the emission intensity of acceptor fluorophore by adding NeuAc which confirmed FRET phenomenon. These spectral FRET approach has been successfully exploited for developing the FRET-based nanosensors in a number of studies (38). The FLIP-SA has been found to be stable in a wide range of the pH and worked well in PBS buffer. Affinity of purified FLIP-SA was confirmed by monitoring the changes in FRET ratio upon binding of different concentrations of NeuAc. In addition, this study authenticate that developed sensor was specific for NeuAc with observation that there was no significant increase in FRET ratio by the addition of other analytes. By utilizing FRET with donor and acceptor molecules different sensors have been developed for detection of varying concentrations of amino acids (22, 29), ions (39, 40), molecular oxygen (35) and plant hormones (41) etc.

Since the prime requirement of the genetically encoded nanosensors is that it should strongly detect the changes in concentration of metabolite in living cell at cellular as well as sub-cellular level in real time to pave the way for better understanding and monitoring the production of the metabolites in the biosynthesis pathway. In the present investigation, the FLIP-SA is capable of real time monitoring of NeuAc flux within single cell and non-invasively. Live cell imaging clearly showed that FLIP-SA nanosensor protein was successfully expressed inside bacterial and yeast cells. Continuous uptake of NeuAc in cellular region, and monitoring of its dynamics with high temporal resolution in real time proved that the nanosensor is effective to work in prokaryotic as well as in eukaryotic cell. Similarly, a range of FRET-based nanosensor to detect the metabolites level in live cells have been successfully characterized by a number of research group (36, 42, 43). Further, developed affinity mutants of the FLIP-SA were used for the measurement of NeuAc level at wide physiological scales. These sensors capable of detection of NeuAc levels from nM to mM range, which fulfilled the main aim of our study to monitor a wide range of NeuAc level at high spatial and temporal resolution. The FLIP-SA will be able to analyze metabolic flux of NeuAc in the biological system. Though, the FLIP-SA has its primary use in the metabolic engineering, it can be used in various other applications. In fermentation industry, it can be used to determine how to manipulate a cellular process in fermentation to control the physiological state of the cells to proceed in a desirable direction. The FLIP-SA can be used for high-throughput screening of *E. coli* mutant library for varied NeuAc production levels.

## Conclusions

Demand of the NeuAc in the global market is increasing continuously because of its use in medical field, infant food additive and cosmetics. Since the production of NeuAc by the traditional methods is very low and the chemical synthesis of the NeuAc is not cost-effective, metabolic engineering approach is being recommended for the biological synthesis of NeuAc. However, understanding of the regulatory mechanism of a metabolite in its metabolic network is the prime requirement for metabolic engineering approach. Metabolic flux analysis of the metabolite is used to identify regulatory step(s) of the metabolic network. However, the metabolic flux analysis requires a tool that can perform real time monitoring of the level of the metabolites in living system. Given this, the present study was conducted to develop a nanosensor that can perform for real time measurement of NeuAc. This nanosensor is based on FRET phenomenon and genetically-encoded. It was successfully used to measure the level of NeuAc in living prokaryotic as well as eukaryotic systems. By using the FLIP-SA nanosensor, the NeuAc concentration can be measured non-invasively in living cells. The FLIP-SP can help in unraveling the regulatory mechanism of the metabolic pathway of NeuAc. Furthermore, the FLIP-SA can be used for screening of *E. coli* mutant library in high-throughput way for varied levels of NeuAc biosynthesis.

## Data Availability Statement

The original contributions presented in the study are included in the article/Supplementary Material, further inquiries can be directed to the corresponding author/s.

## Author Contributions

RN, MO, WS, UF, MM, and AA envisaged and designed the experiments. RN, UF, and AA performed the experiment. RN, MO, IA, MA-M, and AA analyzed the data. RN, MO, WS, MA-M, and AA drafted the manuscript. All authors contributed to the article and approved the submitted version.

## Conflict of Interest

The authors declare that the research was conducted in the absence of any commercial or financial relationships that could be construed as a potential conflict of interest.
