# Supplementary material for: Designing and Development of FRET-Based Nanosensor for Real Time Analysis of N-Acetyl-5-Neuraminic Acid in Living Cells
Source: Front Nutr. 2021 May 31;8:621273. doi: 10.3389/fnut.2021.621273 (PMC8200523; doi:10.3389/fnut.2021.621273)
Supplement: Supplementary file 1 [file Data_Sheet_1.PDF]

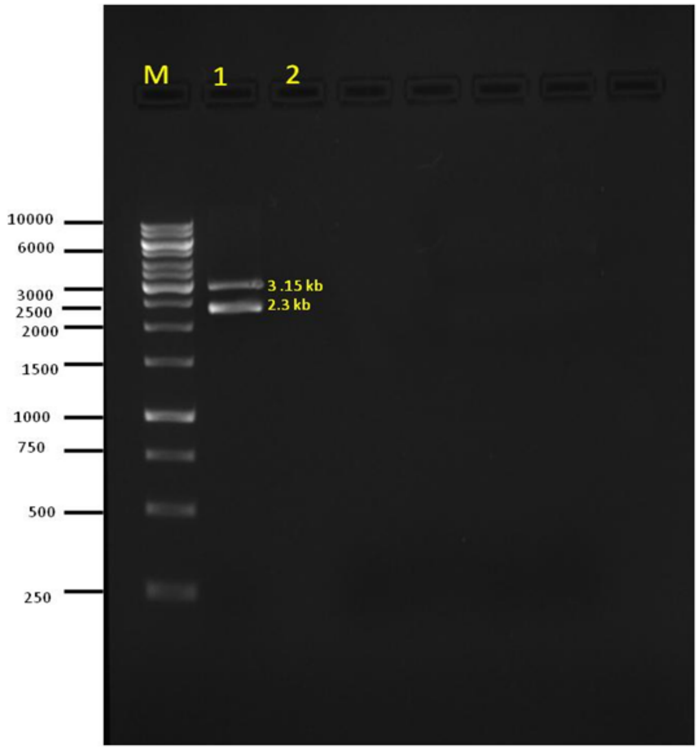

Fig. S1 Restriction digestion of eCFP-SiaP-Venus by *Bam* HI and *Hind* III in pGEM®-T Easy vector. M=DNA marker and Lane 1 Digested products

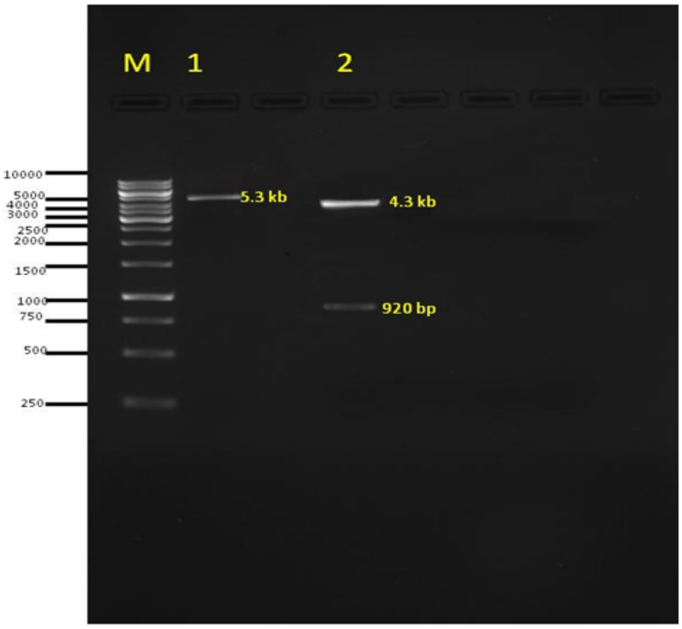

Fig. S2 Restriction digestion pRSET-eCFP-SiaP-Venus by KpnI. SiaP gene (920bp) and pRSET-B vector with eCFP and Venus (4.3 kb);

5'AGTAAAGGAGAAGAACTTTTCACTGGAGTTGTCCCAATTCTTGTTGAATTAGATGGTGATG
TTAATGGGCACAAATTTTCTGTCAGTGGAGAGGGTGAAGGTGATGCAACATACGGAAAACTT
ACCCTTAAATTTATTTGCACTACTGGAAAACTACCTGTTCCATGGCCAACACTTGTCACTACT
TTGACTTGGGGTGTTCAATGCTTTTCAAGATACCCAGATCATATGAAACGGCATGACTTTTT
CAAGAGTGCCATGCCCGAAGGTTATGTACAGGAAAGAACTATATTTTTCAAAGATGACGGGA
ACTACAAGACACGTGCTGAAGTCAAGTTTGAAGGTGATACCCTTGTTAATAGAATCGAGTTA
AAAGGTATTGATTTTAAAGAAGATGGAAACATTCTTGGACACAAATTGGAATACAACTATAT
TTCACACAATGTATACATCACTGCAGACAAACAAAAGAATGGAATCAAAGCTCATTTCAAAA
TTAGACACAACATTGAAGATGGAAGCGTTCAACTAGCAGACCATTATCAACAAAATACTCCA
ATTGGCGATGGCCCTGTCCTTTTACCAGACAACCATTACCTGTCCACACAATCTGCCCTTTCG
AAAGATCCCAACGAAAAGAGAGACCACATGGTCCTTCTTGAGTTTGTAACAGCTGCTGGGAT
TACACATGGCATGGATGAACTATACAA3'

Fig. S3 Complete gene sequences of eCFP without restriction sites.

ATGGTGAGCAAGGGCGAGGAGCTGTCACCGGGGTGGTGCCCATCCTGGTCGAGCTGGACGGCGAC
GTAAACGGCCACAAGTTCAGCGTGTCCGGCGAGGGCGAGGGCGATGCCACCTACGGCAAGCTGAC
CCTGAAGCTCATCTGCACCACCGGCAAGCTGCCCGTGCCCTGGCCCACCCTCGTGACCACCCTCGGC
TACGGCCTCAGTGCTTCGCCCGCTACCCCGACCACATGAAGCAGCACGACTTCTTCAAGTCCGCCATC
CCGAAGGCTACGTCCAGGAGCGCACCATCTTCTTCAAGGACGACGGCAACTACAAGACCCGCGCCG
AGGTGAAGTTCGAGGGCGACACCCTGGTGAACCGCATCGAGCTGAAGGGCATCGACTTCAAGGAG
GACGGCAACATCCTGGGGCACAAGCTGGAGTACAACTACAACAGCCACGACTTCAAGGAGGACGG
CAACATCCTGGGGCACAAGCTGGAGTACAACTACAACAGCCGCACAACATCGAGGACGGCGGCGT
GCAGCTCGCCGACCACTACCAGCAGAACACCCCCATCGGCGACGGCCCCGTGCTGCTGCCCGACAA
CCACTACCTGAGCTACCAGTCCAAGCTGAGCAAAGACCCCAACGAGAAGCGCGATCACATGGTCCT
GCTGGAGTTCGTGACCGCCGCCGG GATCACTCTCGGCATGGACGAGCTGTACAA

Fig. S4. Complete gene sequences of Venus without restriction sites

**cggggtaccccgATTATGACTTGAAATTCGGTATGAATGCTGGAACTTCATCAAATGAATATAAAGCGGCAGAA
ATGTTTGCCAAAGAAGTCAAAGAAAAATCACAGGGTAAAATTGAAATTTCACTTTATCCAAGTTCACAATTA
GGTGATGACCGTGCAATGTTAAAACAATTAAAAGACGGTTCTCTCGACTTTACCTTTGCAGAATCTGCTCGC
TTCCAGCTGTTTTACCCTGAAGCGGCAGTATTTGCCTTACCTTATGTTATTAGCAACTACAATGTTGCACAAA
AAGCCTTATTCGATACAGAATTCGGTAAAGATTTAATTAAAAAAATGGATAAAGATCTTGGCGTGACTTTAC
TTTCCCAAGCTTATAACGGAACTCGCCAAACGACTTCAAATCGTGCAATCAACAGTATTGCAGATATGAAAG
GCTTAAAACTTCGTGTGCCAAATGCAGCAACAAACTTAGCCTATGCTAAATATGTTGGTGCATCACCAACAC
CAATGGCATTTTCTGAAGTTTATCTTGCGTTACAAACCAATGCCGTCGATGGTCAAGAAAACCCGTTAGCAG
CGGTGCAAGCACAAAAATTCTATGAAGTGCAAAAGTTCTTAGCAATGACTAATCATATTTTGAATGACCAAC
TTTATTTAGTAAGCAACGAGACTTATAAAGAACTCCCTGAAGATCTTCAAAAAGTCGTAAAAGATGCTGCCG
AAAATGCAGCAAAATATCACACTAAATTATTCGTAGATGGAGAGAAAGATTTAGTCACATTCTTTGAAAAA
CAAGGCGTGAAAATTACACATCCTGATCTTGTTCCATTTAAAGAATCAATGAAGCCGTATTATGCTGAGTTT
GTAAAACAAACAGATCAAAAAGGTGAATCAGCTTTAAAACAAATTGAAGCAATCAATCCAGAAGGGGGAT
CCCACCACCACCACCAACACcggggtaccccg**

Fig. S5 HI0146 gene sequence without signal peptides and with *KpnI* restriction site

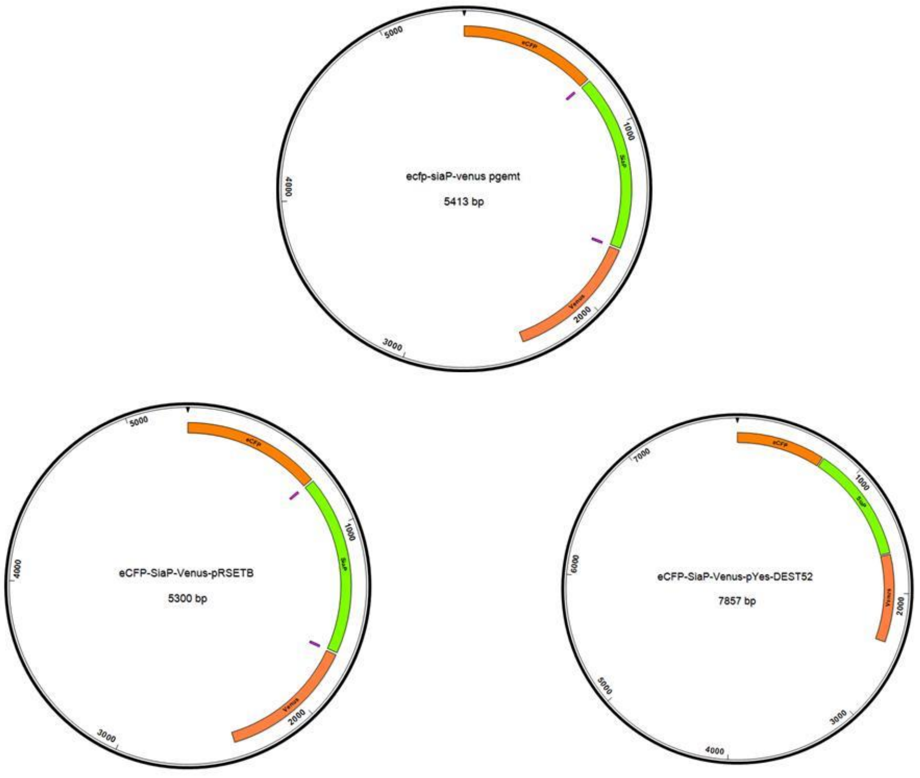

Fig. S6 Construction of FLIP-SA map in cloning (pGEM-T easy) and expression (pRSET-B and pYES-DEST 52) vectors
